# Motivational Factors Influencing Weight Loss in Individuals With a History of Obesity in 2025 at Al-Ahsa, Saudi Arabia

**DOI:** 10.7759/cureus.101503

**Published:** 2026-01-14

**Authors:** Yahya A Almufarrih, Ahmed A Almuthaffar, Khalid A Sharahili, Kawther A Aldandan, Qasem M Aljabr

**Affiliations:** 1 Family Medicine, Al-Ahsa Health Cluster, Ministry of Health, Al-Ahsa, SAU

**Keywords:** al-ahsa, body image, motivational factors, obesity, weight loss

## Abstract

Background: Obesity is defined as an unhealthy accumulation of body fat that increases the risk of chronic diseases. Motivation is essential for weight loss. It can be due to intrinsic factors (e.g., enhanced health) or extrinsic factors (e.g., physical appearance or social approval). The aim of this study was to identify the motivational factors influencing weight loss in individuals with a history of obesity in 2025 at Al-Ahsa, Saudi Arabia.

Methods: A descriptive, cross-sectional, questionnaire-based study was conducted to identify the motivational factors influencing weight loss in individuals with a history of obesity in 2025 at Al-Ahsa, Saudi Arabia. A stratified sample involved 367 participants who were obese and lost 5% or more of their weight in the last six months. IBM SPSS Statistics for Windows, Version 27.0 (IBM Corp., Armonk, New York, United States), was used to analyze the data, and the chi-squared test was used to correlate the motivational factors with sociodemographic characteristics.

Results: The study included 367 participants: more than half of them were female (219, 59.7%), more than one-quarter (108, 29.4%) were aged between 30 and 40 years, more than half of them (218, 59.4%) had class I obesity, and an additional 149 (40.6%) had class II or III obesity. The mean motivational factors for weight loss among participants were as follows: enhance overall physical wellness (277, 75.5%), promote physical appearance (172, 46.9%), desire to fit into attire (143, 39%), and strengthen self-efficacy and self-image (134, 36.5%). The motivational factors for weight loss are significantly influenced by various sociodemographic characteristics.

Conclusion: This study concluded that obese adults in Al-Ahsa are primarily motivated to improve physical wellness, followed by physical appearance, fitting into attire, and self-esteem. These motivations are significantly influenced by sociodemographic factors. Therefore, focused, simple, and clear educational media campaigns are recommended to explain the benefits of weight loss in the community.

## Introduction

Obesity is characterized by an abnormal or excessive accumulation of body fat that poses a risk to health. It is strongly associated with an increased likelihood of developing chronic conditions, such as cardiovascular illnesses, type 2 diabetes, and mental health disorders, as well as puts a significant burden on healthcare systems [1,2]. According to the 2019 National Health Information Survey, 20.2% of adults in Saudi Arabia were obese, and 38.2% were overweight. The prevalence of obesity was higher among women (21.4%) compared to men (19.2%) [3].

Motivation is essential in weight loss, with individuals displaying different levels of intrinsic and extrinsic motivation. Literature demonstrates that intrinsic motivation, such as the inspiration for enhanced health and well-being, generally proves more effective in maintaining long-term weight loss than extrinsic motivators like social pressure or aesthetic considerations [4,5]. Nonetheless, numerous individuals continue to seek weight loss for extrinsic motivations, like enhancing physical appearance or achieving social approval [6,7]. Self-determination theory posits that individuals are more inclined to adhere to behavioral therapies when they perceive a sense of control over their weight reduction journey [8,9]. Factors such as psychological discomfort, weight stigma, and emotional issues may hinder success and lead to elevated dropout rates [10,11]. Although some studies highlight the significance of motivational components, primary care programs frequently emphasize nutrition and physical activity advice over customized psychosocial support [12]. Patients often express a difference between standardized interventions and their personal needs, including the management of emotional causes or motivating factors [13,14]. Differences between genders in weight loss motivation have been noted, with men mostly addressing health-related issues as key motivators, while women often highlight aesthetic and social aspects [15,16].

However, the degree to which these factors interact to influence long-term outcomes, including sustainable weight maintenance, physical well-being, and the reduction of chronic disease risk, remains insufficiently investigated [17]. Despite substantial advancements in obesity research, limited attention has been given to the role of motivational factors within primary healthcare settings and their influence on weight loss outcomes. This study aims to identify the motivational factors influencing weight loss in individuals with a history of obesity in 2025 at Al-Ahsa, Saudi Arabia. This research seeks to answer the question "What are the key motivational factors influencing weight loss attempts among adults with a history of obesity?" and to provide evidence-based recommendations for enhancing obesity management by identifying key motivators.

## Materials and methods

Study design

A descriptive cross-sectional questionnaire-based study was conducted to evaluate the motivational factors influencing weight loss in individuals with a history of obesity in 2025 at Al-Ahsa, Saudi Arabia. The study was carried out among primary healthcare facilities in the rural and urban areas of Al-Ahsa, Saudi Arabia.

Study population

The study population involved adult people residing in Al-Ahsa, Saudi Arabia, who were obese (BMI: ≥30) and lost 5% of their weight in the last six months. To be included, participants must have achieved or attempted a weight loss of 5% or more of their initial body weight within the preceding six months. Individuals were excluded if they were pregnant, had severe mental or cognitive illnesses that impair judgment (such as dementia or schizophrenia), or had conditions where weight loss is medically inadvisable (e.g., advanced cancer, terminal disease). Participants who refused to provide the weight and height data required for BMI calculation were also excluded.

Sample size and technique

A stratified random sampling technique was used to select participants from a registry of 60,000 adult patients classified as obese and enrolled in team-based care in Al-Ahsa. Strata were defined based on age group (18-35, 36-55, 56+) and gender (male, female).

Each patient was assigned a unique ID, and then random numbers were generated for the selected final sample.

The minimum required sample size was calculated using Raosoft (Raosoft Inc., Seattle, Washington, United States) assuming a 95% confidence level, an estimated proportion (p) of 50%, and a 5% margin of error (d). Based on these parameters, the minimum sample size was determined to be 382. We added 10% to compensate for expected non-response. So the final calculated sample size was 420. However, only 367 were included in the final analysis, with a response rate of 87.4%.

Data collection

Data were collected through a structured, validated questionnaire (Appendix 1). The research team conducted the interviews following a standardized script to ensure consistency. Each call lasted approximately 10-15 minutes. Participants were contacted using the phone numbers listed in the healthcare database, and verbal informed consent was obtained prior to the interview.

Study tool

The data were collected using a questionnaire structured by the researchers to assess the self-reported reasons for attempting weight loss among obese adults. Each item is rated on a 3-point Likert scale (1=not at all; 2=somewhat; 3=a lot), indicating the extent to which each factor serves as a motivator for weight loss. The instrument underwent expert content validation by three family medicine specialists and a biostatistician, and the pilot study revealed a Cronbach's alpha of 0.9 indicating good readability (Appendix 2).

Data analysis

Data were collected, entered into an Excel sheet (Microsoft Corporation, Redmond, Washington, United States), and coded and analyzed using IBM SPSS Statistics for Windows, Version 27.0 (IBM Corp., Armonk, New York, United States). The data were presented as frequency and percentage in tables or figures. The data were tested using the Shapiro-Wilk test and the Kolmogorov-Smirnov test and followed a normal distribution. The chi-squared test was used to find the relationship between the demographic characteristics (sex, age, education, have children at home, BMI, self-perceived weight status, and recommended weight loss by healthcare providers) and motivational factors influencing weight loss. A p-value of ≤0.05 is considered statistically significant.

Ethical approval

Ethical approval for this study was granted by the Institutional Review Board (IRB) of Prince Saud Bin Jalawi Hospital (approval number: 14-EP-2025) on June 25, 2025. The research was conducted in strict accordance with the principles of the Declaration of Helsinki. Prior to participation, all individuals were informed of the study's objectives and provided informed consent. The confidentiality of participant data was maintained throughout the collection process. Participants were also explicitly informed of their right to withdraw at any time without coercion, and it was guaranteed that no individual information would be shared with third parties.

## Results

Table 1 shows that the study included 367 participants: more than half of them were female (219, 59.7%), and more than one-quarter (108, 29.4%) were aged between 30 and 40 years. More than two-thirds (245, 66.8%) held less than a college degree, and a majority (256, 69.8%) had children at home. Regarding weight, more than half of them (218, 59.4%) had class I obesity, and an additional 149 (40.6%) had class II or III obesity (BMI≥35 (kg/m^2^)). Regarding self-perceived weight, most of the participants (310, 84.5%) considered themselves at least a little overweight, and more than half of them (231, 62.9%) reported having received a weight loss recommendation from a healthcare provider.

**Table 1:** Participants' sociodemographic characteristics (n=367)

Sociodemographic characteristics		Percentage	Frequency
Sex	Male	148	40.3
	Female	219	59.7
Age (in years)	Less than 30	57	15.5
	30-40	108	29.4
	41-50	75	20.4
	51-60	71	19.3
	More than 60	56	15.3
Education	Less than a college degree	245	66.8
	≥ college degree	122	33.2
Any children at home	Yes	256	69.8
	No	111	30.2
BMI (kg/m^2^)	30-34.9	218	59.4
	35-39.9	94	25.6
	≥40	55	15
Self-perceived weight status	Very overweight	96	26.2
	Somewhat overweight	120	32.7
	A little overweight	94	25.6
	Not overweight	57	15.5
Healthcare provider ever recommended weight loss	Yes	231	62.9
	No	136	37.1

Table 2 reveals that the motivations for weight loss among participants were improving health (277, 75.5%). This is followed by improving physical appearance, which is a strong motivator for 172 (46.9%). The desire to fit into attire (143, 39%) and strengthen self-efficacy and self-image (134, 36.5%) are also significant. Less prominent, but still relevant, is the goal of emotional balance and joy, cited by 126 (34.3%). Also, attentive partner/spouse (113, 30.8%), inspire others (98, 26.7%), attentive partner/spouse (93, 25.3%), and maximize professional productivity and output (88, 24%) consistently rank as the least impactful factors driving weight loss efforts.

**Table 2:** Reasons for trying to lose weight (n=367)

Items		Not at all	Somewhat	A lot
Enhance overall physical wellness	N	43	47	277
	%	11.7	12.8	75.5
Promote my physical appearance	N	121	74	172
	%	33	20.2	46.9
Emotional balance and joy	N	155	86	126
	%	42.2	23.4	34.3
Strengthen self-efficacy and self-image	N	137	96	134
	%	37.3	26.2	36.5
Attentive partner/spouse	N	218	56	93
	%	59.4	15.3	25.3
Enhancing the relationships	N	182	72	113
	%	49.6	19.6	30.8
Maximize professional productivity and output	N	201	78	88
	%	54.8	21.3	24
Fit into my attire	N	159	65	143
	%	43.3	17.7	39
Inspire others	N	202	67	98
	%	55	18.3	26.7

Table 3 demonstrates that motivations for weight loss are significantly influenced by various sociodemographic characteristics, with age and self-perceived weight status emerging as the most consistent predictors across nearly all reasons. Specifically, improving health is strongly associated with BMI (p=0.004), self-perceived weight status (p<0.001), and healthcare provider recommendations (p<0.001). Physical appearance as a motivator is significantly linked to age (p<0.001), education (p=0.001), and self-perceived weight status (p<0.001). Similarly, emotional balance and joy is significantly influenced by age (p<0.001), education (p=0.017), and self-perceived weight (p<0.001), while feeling better about oneself varies significantly by age (p=0.022), education (p=0.008), and self-perceived weight (p<0.001). The desire to fit into attire shows the broadest associations, significantly varying by sex (p=0.005), age (p<0.001), education (p=0.008), and self-perceived weight status (p<0.001). Less universally impactful, enhancing the relationships is influenced by age (p=0.034) and self-perceived weight (p=0.002), and maximize professional productivity and output is linked to age (p<0.001), self-perceived weight (p=0.001), and a healthcare provider's recommendation (p=0.040). Conversely, attentive partner/spouse as a motivation shows no significant associations with any sociodemographic characteristic, and inspire others is only significantly linked to self-perceived weight status (p=0.038).

**Table 3:** Relationship between participants' sociodemographic characteristics and reasons for trying to lose weight *: significant; χ2 value: chi-squared value; HCPs: healthcare professionals

Causes		Sex	Age	Education	Any children at home	BMI (kg/m^2^)	Self-perceived weight status	Recommended weight loss by HCPs
Enhance overall physical wellness	χ2 value	2.43	10.865	3.544	4.552	15.211	113.796	52.478
	p-value	0.297	0.209	0.17	0.103	0.004*	<0.001	<0.001
Promote my physical appearance	χ2 value	5.162	58.831	13.756	3.369	8.672	47.910	4.566
	p-value	0.297	<0.001	0.001*	0.186	0.070	<0.001	0.102
Emotional balance and joy	χ2 value	4.607	43.062	8.142	1.457	2.981	26.839	3.988
	p-value	0.100	<0.001	0.017*	0.483	0.561	<0.001	0.136
Strengthen self-efficacy and self-image	χ2 value	0.254	17.871	9.642	2.877	7.167	31.489	3.778
	p-value	0.881	0.022*	0.008*	0.237	0.127	<0.001	0.151
Attentive partner/spouse	χ2 value	1.646	10.142	2.622	3.976	2.048	10.344	1.323
	p-value	0.439	0.255	0.270	0.137	0.727	0.111	0.516
Enhancing the relationships	χ2 value	0.361	16.638	0.653	0.671	3.878	20.380	4.643
	p-value	0.835	0.034*	0.722	0.715	0.423	0.002*	0.098
Maximize professional productivity and output	χ2 value	2.592	33.093	5.481	4.536	3.417	21.868	6.431
	p-value	0.274	<0.001	0.065	0.104	0.491	0.001*	0.040*
Fit into my attire	χ2 value	10.747	35.859	9.688	0.062	5.449	37.594	4.017
	p-value	0.005*	<0.001	0.008*	0.969	0.244	<0.001	0.134
Inspire others	χ2 value	1.923	11.716	4.619	2.523	0.763	13.349	2.082
	p-value	0.382	0.164	0.099	0.283	0.943	0.038*	0.353

**Figure 1:**
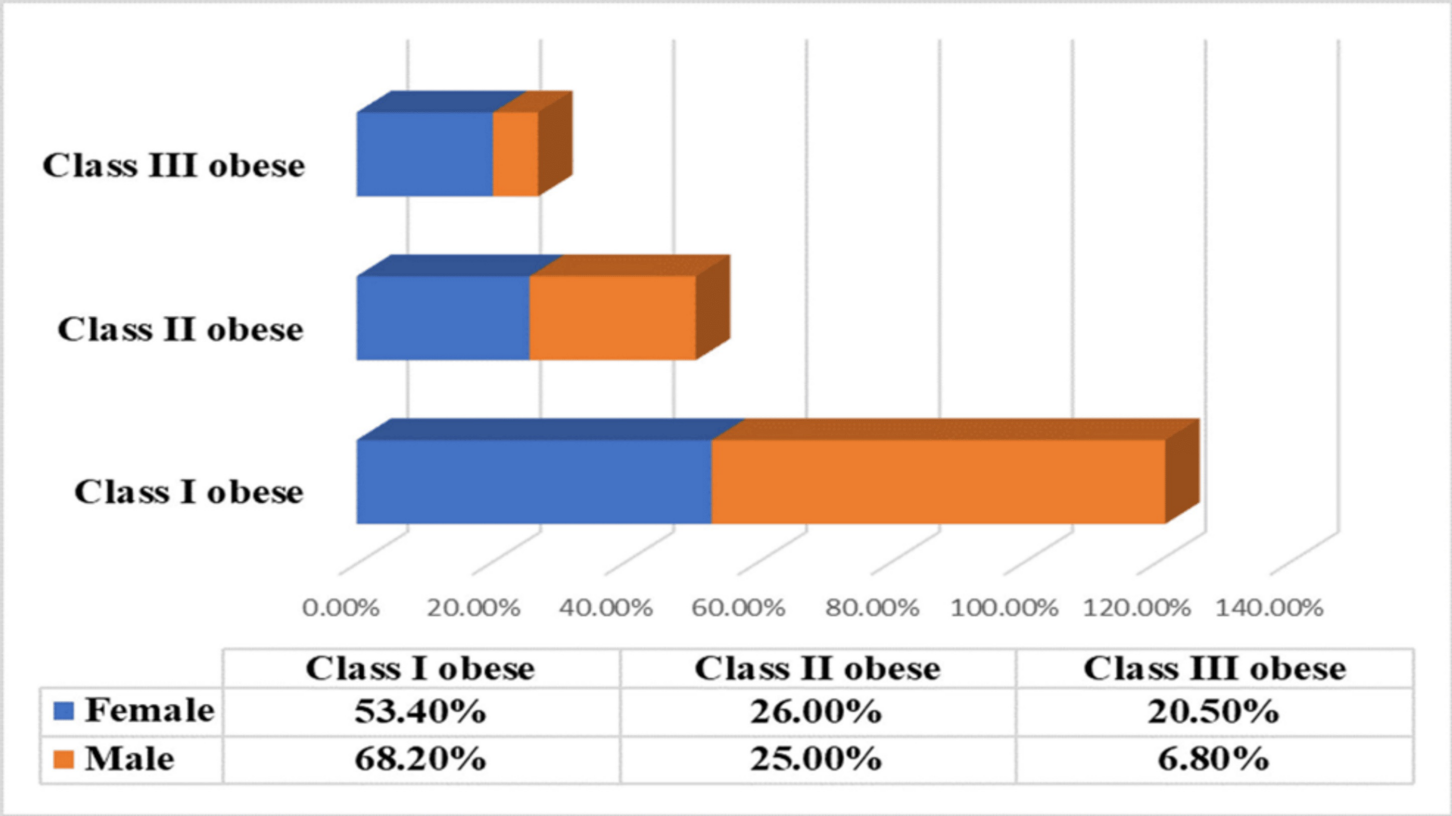
Weight status (BMI) according to gender (p<0.001) P<0.001 is considered statistically significant.

Figure 1 demonstrates a statistically significant gender difference in the distribution of obesity classes (p<0.001). It shows that class I obesity (BMI 30-34.9 (kg/m^2^)) is proportionally more common among obese males (101, 68.2%) compared to females (117, 53.4%). Conversely, class III obesity (BMI ≥ 40(kg/m^2^)) is more prevalent in females (45, 20.5%) versus obese males (10, 6.8%), while class II obesity (BMI 35-39.9 (kg/m^2^)) remains relatively similar between both genders (26% for females and 25% for males).

**Figure 2:**
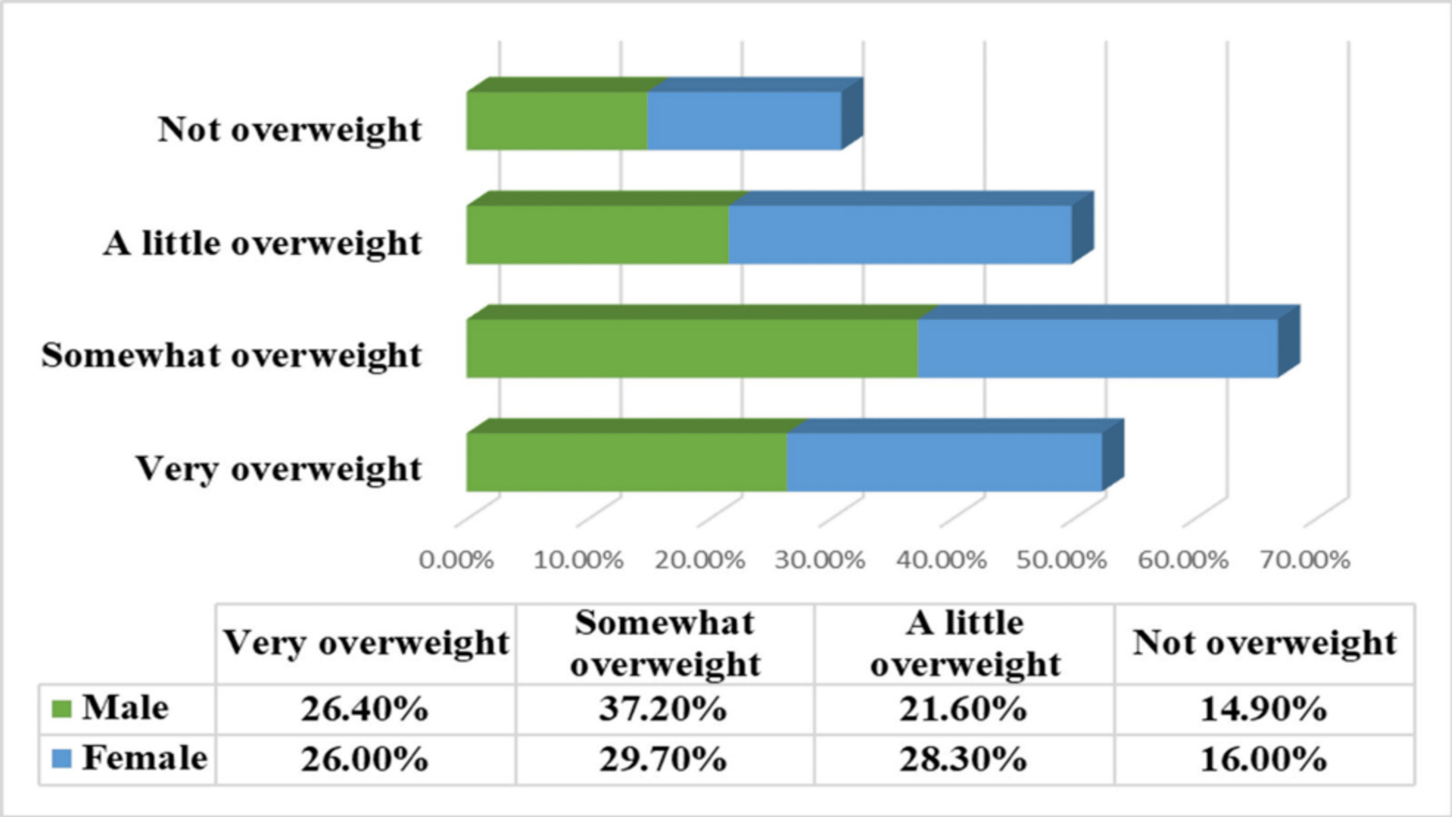
Perceived weight status according to gender (p=0.372) P=0.372 is considered statistically not significant.

Figure 2 illustrates that there is no statistically significant difference in perceived weight status between males and females (p=0.372). Specifically, similar proportions of males (39, 26.4%) and females (57, 26%) perceive themselves as very overweight. While a slightly higher percentage of males (55, 37.2%) identify as somewhat overweight compared to females (65, 29.7%), females (62, 28.3%) more often perceive themselves as a little overweight than males (32, 21.6%). The proportion of those feeling not overweight also remains very similar between males (22, 14.9%) and females (35, 16%).

## Discussion

In recent decades, there has been a relentless increase in belt sizes coupled with a decline in physical activity across the globe. The Kingdom of Saudi Arabia serves as a prime example of this phenomenon; it currently holds one of the highest obesity rates worldwide [18]. The report further states that between 70% and 75% of adults in Saudi Arabia are classified as overweight, with approximately 35% categorized as obese [19,20]. The obesity rate among men ranges from 30% to 40%, while 50-60% of women in Saudi Arabia are classified as overweight. This issue of obesity also extends to the younger population, where one-quarter of Saudi boys are overweight and over one-third of girls are similarly affected [19]. Moreover, national studies highlight that a considerable percentage of the Saudi population does not engage in any form of physical activity nor adhere to the daily exercise guidelines, which poses a considerable risk factor for obesity [21].

Enhancing one's health is motivated by the desire to maintain personal well-being, to adopt a healthy lifestyle, and to address concerns regarding current illnesses and health threats. In our study, about three-quarters (277, 75%) mentioned that the main reason to lose weight was to improve their physical wellness. This finding contrasts with a Saudi study conducted among female university students, where only 14% identified health enhancement as a reason for engaging in weight control behaviors [22]. Similarly, another Saudi study conducted in Riyadh among high school students found that health concerns ranked lowest among the motivations for physical activity [23]. This variation may be attributed to a shift in motivational salience; as individuals age, the onset of obesity-related comorbidities, such as cardiovascular diseases, transforms health from a secondary concern into a primary necessity. In contrast, younger populations are often influenced by higher levels of social and aesthetic pressure, prioritizing physical appearance over long-term wellness.

Our findings reflect a more health-driven perspective among the participants, possibly influenced by age-related health risks, while the two previous studies were conducted among those of younger age, who are more concerned with general appearance rather than their health. However, we did not find a statistically significant difference between health concerns and different age categories. Furthermore, health concern was statistically significant with the weight of participants, whereas those considered overweight and obese were more likely to be motivated by health concern. This finding was consistent with a study conducted in Thailand, revealing that health concerns were more pronounced among the "moderate to severe obesity" category [17].

Promoting physical appearance and fitting into clothes were the next most frequently reported motivations for weight loss among our participants, particularly prominent among younger age groups and females. A statistically significant association was observed between gender and the desire to fit into clothes, as well as between age categories and both improving appearance and fitting into clothes. These findings align with previous studies indicating that women frequently place greater importance on appearance-related factors, such as the desire to fit into clothing, rather than health, when it comes to their motivations for weight loss [18]. Additionally, research conducted by Mroz et al. revealed that motivations related to personal appearance could, over time, lead to less favorable weight outcomes in comparison to health-oriented objectives [24]. In particular, adolescent girls often face heightened pressure and influence from both media and peers to attain a slim and well-defined body that conforms to socially constructed standards of beauty [6].

Moderately rated motivations for weight loss included emotional balance and joy (126, 36%), strengthen self-efficacy and self-image (134, 34%), and enhancing social life (113, 30%). Inconsistently, a study by Md-Yasin et al. [25] reported that the desire to feel and look good and to improve one's relationships were among the least cited motivations in their sample, accounting for only 12% and 3% of participants, respectively. Additionally, in our study, these three motivations were more likely to be strongly related to younger middle-aged groups, suggesting a possible generational change in the perception of weight loss evolving from a focus on health to also include aspects of self-esteem and social functioning. This highlights the importance of adapting a personalized approach in weight management interventions, suggesting that programs for younger or middle-aged adults should incorporate aspects that address emotional well-being and social confidence alongside physical health [26].

The least rated motivations for weight loss were to inspire others (98, 26%), to be a better spouse or partner (93, 25%), and to improve productivity (88, 24%). Moreover, we found a statistically significant association between the motivation to improve job performance and age groups, with this factor being most prevalent among younger participants. The strongest motivation was reported in the 30-40-year age group (34.3%), closely followed by participants under 30 years old (29.8%). These findings were aligned with a systematic review study, which revealed that motivations associated with job performance were less frequently documented in various studies, with health and appearance being the most commonly cited factors, but motivations related to work were more widespread among younger adult demographics [27].

In our study, we observed a statistically significant association between self-perceived weight status and nearly all weight loss motivators (seven out of eight). This finding underscores the powerful influence of subjective body image on individuals' motivation to lose weight. Participants who perceived themselves as overweight were more likely to report motivations such as improving health, enhancing appearance, fitting better into clothes, and increasing self-esteem. These results align with previous studies suggesting that perceived weight status is often a stronger predictor of weight-related behaviors than actual BMI. For example, Johnson-Taylor et al. [28] found that individuals who consider themselves overweight are more likely to attempt weight loss, regardless of their actual weight. Similarly, Yaemsiri et al. [29] reported that self-perception of overweight status significantly influences weight control practices in both men and women. Education programs and campaigns, such as the National Campaign Against Overweight and Obesity, should be carried out through different media to explain the benefits of weight loss.

Strengths and limitations of the study

The study's strengths lie in its focus on a clinically relevant population (those with initial weight loss), the use of a valid questionnaire, a large sample size, and a stratified sample based on age and gender. However, its limitations include a cross-sectional design that prevents establishing causality or long-term trends, a reliance on self-reported data susceptible to recall and social desirability bias, and limited generalizability due to its specific geographical focus on Al-Ahsa, Saudi Arabia.

Clinical implications and future recommendations

Based on the study's findings, focused, simple, and clear education programs and campaigns should be carried out. Clinicians should emphasize physiological benefits, such as improved metabolic markers and energy levels, while tailoring counseling to specific sociodemographic profiles, such as addressing self-image in younger populations and chronic disease prevention in older groups. To support long-term success, incorporating cognitive-behavioral strategies is essential to bolster self-efficacy and self-image. Consequently, policymakers should launch targeted media campaigns that visually link weight loss to these community-specific motivators. Future research should utilize longitudinal designs to identify which factors best predict sustainable weight maintenance beyond six months, alongside qualitative explorations to further understand the cultural nuances of sociodemographic influences. 

## Conclusions

This study concludes that among obese adults in Al-Ahsa, Saudi Arabia, who have recently lost weight, the primary motivation for doing so is overwhelmingly health-related, improving physical appearance, the desire to fit into clothes, and enhancing self-esteem. The research further establishes that these motivations are significantly influenced by sociodemographic characteristics such as age and gender.

## Appendices

Appendix 1

**Table 4:** Sociodemographic characteristics

Sociodemographic characteristics		Answer
Sex	Male	
	Female	
Age (in years)	Less than 30	
	30-40	
	41-50	
	51-60	
	More than 60	
Education	Less than a college degree	
	≥ college degree	
Any children at home	Yes	
	No	
BMI (kg/m^2^)	30-34.9	
	35-39.9	
	≥40	
Self-perceived weight status	Very overweight	
	Somewhat overweight	
	A little overweight	
	Not overweight	
Healthcare provider ever recommended weight loss	Yes	
	No	

Appendix 2

**Table 5:** Reasons for trying to lose weight

Reasons	Not at all	Somewhat	A lot
Enhance overall physical wellness			
Promote my physical appearance			
Emotional balance and joy			
Strengthen self-efficacy and self-image			
Attentive partner/spouse			
Enhancing the relationships			
Maximize professional productivity and output			
Fit into my attire			
Inspire others			
